# Targeting ROS and cPLA2/COX2 Expressions Ameliorated Renal Damage in Obese Mice with Endotoxemia

**DOI:** 10.3390/ijms20184393

**Published:** 2019-09-06

**Authors:** Jia-Feng Chang, Jih-Chen Yeh, Chun-Ta Ho, Shih-Hao Liu, Chih-Yu Hsieh, Ting-Ming Wang, Shu-Wei Chang, I-Ta Lee, Kuo-Yang Huang, Jen-Yu Wang, Wei-Ning Lin

**Affiliations:** 1 Division of Nephrology, Department of Internal Medicine, En Chu Kong Hospital, New Taipei City 237, Taiwan (J.-F.C.) (C.-Y.H.); 2 Renal Care Joint Foundation, New Taipei City 220, Taiwan (J.-C.Y.) (C.-T.H.); 3 Department of Nursing, Yuanpei University of Medical Technology, Hsinchu 300, Taiwan; 4 Graduate Institution of Biomedical and Pharmaceutical Science, College of Medicine, Fu Jen Catholic University, New Taipei City 242, Taiwan; 5 Division of Nephrology, Department of Internal Medicine, Shuang Ho Hospital, Taipei Medical University, New Taipei City 235, Taiwan; 6 Department of Dentistry, Far Eastern Memorial Hospital, New Taipei City 220, Taiwan; 7 Division of Pathology, En-Chu-Kong Hospital, New Taipei City 237, Taiwan; 8 Department of Orthopaedic Surgery, School of Medicine, National Taiwan University, Taipei 106, Taiwan; 9 Department of Orthopaedic Surgery, National Taiwan University Hospital, Taipei 106, Taiwan; 10 Department of Civil Engineering, National Taiwan University, Taipei 106, Taiwan; 11 School of Dentistry, College of Oral Medicine, Taipei Medical University, Taipei 110, Taiwan; 12 Graduate Institute of Pathology and Parasitology, National Defense Medical Center, Taipei 114, Taiwan

**Keywords:** obese kidney fibrosis, endotoxemia, ROS, cPLA2 and COX-2

## Abstract

Obesity is associated with metabolic endotoxemia, reactive oxygen species (ROS), chronic inflammation, and obese kidney fibrosis. Although the fat–intestine–kidney axis has been documented, the pathomechanism and therapeutic targets of obese kidney fibrosis remain unelucidated. To mimic obese humans with metabolic endotoxemia, high-fat-diet-fed mice (HF group) were injected with lipopolysaccharide (LPS) to yield the obese kidney fibrosis–metabolic endotoxemia mouse model (HL group). Therapeutic effects of ROS, cytosolic phospholipases A2 (cPLA2) and cyclooxygenase-2 (COX-2) inhibitors were analyzed with a quantitative comparison of immunohistochemistry stains and morphometric approach in the tubulointerstitium of different groups. Compared with basal and HF groups, the HL group exhibited the most prominent obese kidney fibrosis, tubular epithelial lipid vacuoles, and lymphocyte infiltration in the tubulointerstitium. Furthermore, inhibitors of nonspecific ROS, cPLA2 and COX-2 ameliorated the above renal damages. Notably, the ROS-inhibitor-treated group ameliorated not only oxidative injury but also the expression of cPLA2 and COX-2, indicating that ROS functions as the upstream signaling molecule in the inflammatory cascade of obese kidney fibrosis. ROS acts as a key messenger in the signaling transduction of obese kidney fibrosis, activating downstream cPLA2 and COX-2. The given antioxidant treatment ameliorates obese kidney fibrosis resulting from a combined high-fat diet and LPS—ROS could serve as a potential therapeutic target of obese kidney fibrosis with metabolic endotoxemia.

## 1. Introduction

The worldwide increase in the prevalence of obesity has occurred in parallel with an increasing prevalence of chronic diseases, including diabetes mellitus, hypertension, cardiovascular diseases and chronic kidney disease (CKD) [1]. In light of this, the focus of World Kidney Day 2017 is kidney disease and obesity. It is found that the increase of BMI positively related to the presence and development of low estimated glomerular filtration rate and the incidence of end-stage renal disease [2]. Even metabolically healthy obesity is not a harmless condition—the phenotype of obese humans, no matter the abnormalities of metabolism, is reversely correlated with renal function [3]. In addition, the fat–intestine–kidney axis has a pivotal position in the mechanism of obese kidney disease, involving metabolic endotoxemia (ME) from intestinal dysbiosis, reactive oxygen species (ROS), systemic inflammation, and progressive renal fibrosis [4]. The rupture of the epithelial barrier results in the translocation of endotoxin and changes in the microbiome, which correlate to the occurrence of inflammation [5]. The evaluation of blood bacterial DNA shows that *Proteobacteria phylum*, *Gammaproteobacteria* class, and *Enterobacteriaceae* and *Pseudomonadaceae* families are more abundant in the CKD group compared with healthy controls [6]. Furthermore, there is elevated plasma lipopolysaccharide (LPS)-binding protein in hemodialysis patients with metabolic syndrome and obesity [7]. These studies suggested that LPS, also called endotoxin, functioned as a linker between the gut microbiome and CKD.

Emerging evidence indicates that prostaglandin (PG) pathways intricately interact with diabetes, metabolic syndrome, CKD progression, and cardiovascular events [8]. In a fatty rat model, renal cyclooxygenase-2 (COX-2) protein expression was accentuated and correlated with metabolic abnormalities [9]. Furthermore, a myriad of studies showed inhibition of cytoplasmic phospholipase A2 (cPLA2) and downstream signals attenuate renal injury [10,11,12,13]. Initiation of the PG signaling pathway usually occurs with the release of arachidonic acids from phospholipids within membranes by cPLA2. The subsequent conversion of arachidonic acid into PG is facilitated by COX-2, leading to inflammation and fibrosis in the kidney. Indeed, compared with healthy controls, renal tissues in CKD groups exhibit higher expressions of fibroblasts and the *COX-2* gene [14]. Although cPLA2 and eicosanoids contributed to renal oxidative stress, inflammation, and end-organ damage [15], eradication of bone-marrow-derived cPLA2 attenuated the eicosanoid storm and renal fibrosis [16].

Intracellular redox imbalance plays important roles in the pathogenesis of CKD. Plasma cells of CKD patients show activation of NF-κB and up-regulated expression of pro-inflammatory and pro-oxidant genes, as well as down-regulation of Nrf2-associated antioxidant gene expression [14]. Similarly, the nephrotoxic agent, aristolochic acid I, increases protein abundance of the NADPH oxidase subunits NOX4, p47^phox^, p22^phox^ and 3-nitrotyrosine in rats within 8 to 24 weeks [17]. Moreover, upregulation of intracellular ROS promotes the expression of inflammatory genes, including *cPLA2* and *COX-2* [18,19]. In fact, ameliorating oxidative stress via modulating forkhead box class O1 (FOXO1) expression attenuated high glucose-induced renal proximal tubular cell injury [20].

Despite previously documented implications, the pathomechanism and therapeutic targets of obese kidney fibrosis (OKF) remain unelucidated. To mimic obese humans with ME, we developed a combined high-fat-diet-fed (HF) and lipopolysaccharide (LPS)-treated mouse model to explore inflammatory signaling pathways of OKF. We hypothesized that OKF is involved in ROS generations, activating downstream cPLA2 and COX-2, resulting in progressive tubulointerstitial fibrosis. Thus, ROS could serve as a potential therapeutic target of OKF. Quantitative comparison of immunohistochemical (IHC) staining and morphometric approach were used to test this hypothesis.

## 2. Results

### 2.1. HF Mice with ME Exhibit the Most Prominent Renal Fibrosis, Depositions of Lipid Vacuoles in Tubular Epithelium, and Lymphocyte Infiltration in Tubulointerstitium

**Figure 1:**
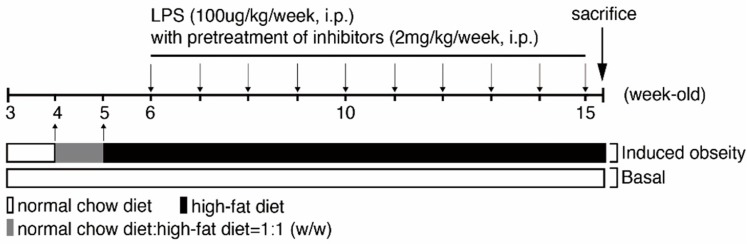
Model of obese kidney fibrosis with metabolic endotoxemia (OKF-ME). Mice were randomized into three groups: normal chow-fed control group (basal group), high-fat diet-fed group (HF group), and high-fat diet-fed group with lipopolysaccharides (LPS) treatment (HL group). The HL group was induced with LPS (100 μg/kg/week intraperitoneal (i.p.) injection) as the working model of OKF-ME.

**Figure 2:**
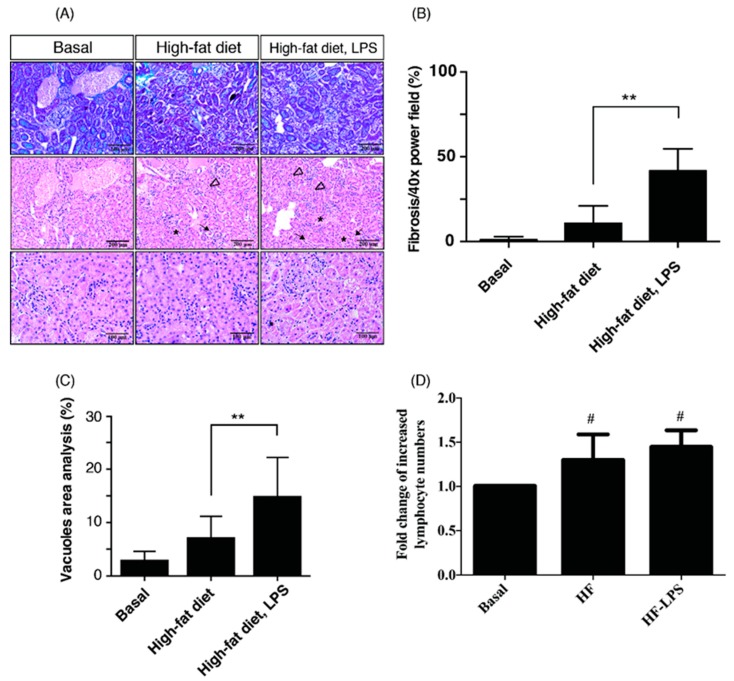
HF mice with ME exhibit the most prominent renal fibrosis, depositions of lipid vacuoles in tubular epithelium, and lymphocyte infiltration in tubulointerstitium. (**A**) Mice were scarified at the end of treatment, and kidney tissues were extracted for histological staining (Haematoxylin and Eosin and Masson’s trichrome staining). Compared with the basal group and HF mice, the HL group exhibited the most prominent renal fibrosis, depositions of lipid vacuoles in tubular epithelium, and lymphocyte infiltration in the tubulointerstitium. Black hollow triangles indicate shedding of tubules. Black arrows indicate infiltration of lymphocytes. Stars indicate lipid vacuoles. Scale bars in the panels are 200 μm or 100 μm as indicated. Quantification analyses were performed by ImageJ software for (**B**) fibrosis area (**C**) lipid vacuoles and (**D**) lymphocyte number. Data are expressed as mean ± SD (*n* = 8); ** p < 0.01, to compare the differences between the two indicated groups. *#* *p* < 0.05, compared with the basal group.

Recent evidence indicates that adipose tissue inflammation in obesity and metabolic syndrome is not strictly a macrophage-dependent phenomenon. Lymphocytes, especially T cells, infiltrate the adipose tissue and mediate the inflammatory response. The infiltration of T effector cells precedes the accumulation of macrophages in adipose tissue and reducing regulatory T cells during obesity are thought to contribute to the development of adipose tissue inflammation [21]. Deposition of lipid vacuoles (LV) in proximal tubular epithelial cells are the typical features of fatty kidneys [22,23], and renal fibrosis is a hallmark and common outcome across all kinds of progressive CKD [24]. To investigate human obese kidney fibrosis with metabolic endotoxemia, we used an experimental rodent model, fed a high-fat diet with LPS injection (called the OKF-ME model) (Figure 1). In accordance with previous studies [22,23], our high-fat diet course induced shedding of renal tubule epithelial cells, tubular LV deposition and increased lymphocyte infiltration in renal tubulointerstitium with or without LPS treatment at week 15 (Figure 2A). Compared with the basal group and HF mice, the HL group exhibited the most prominent fibrosis (Figure 2B), tubular LV accumulation (Figure 2C), and lymphocyte infiltration in tubulointerstitium (Figure 2D). Current data demonstrate that HF and ME result in not only renal tubule injury but also tubulointerstitial inflammation and fibrosis.

### 2.2. Inhibitors of ROS, cPLA2, and COX-2 Attenuate the Tubulointerstitial Fibrosis in HF Mice with ME

**Figure 3:**
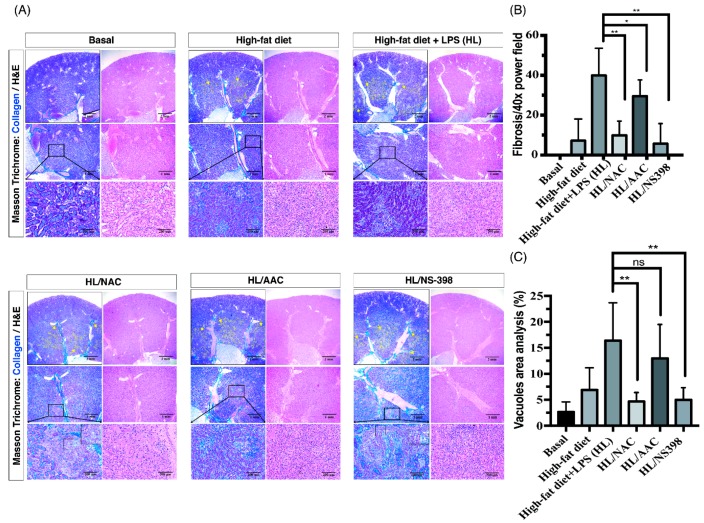
Inhibitors of reactive oxygen species (ROS), cPLA2, and COX-2 attenuate the tubulointerstitial fibrosis in HF mice with ME. (**A**) The HL group exerts the most significant collagen deposition and tubulointerstitial fibrosis in the juxtamedullary to medullar regions. In corresponding fibrotic regions, the HL/N-acetylcysteine (NAC) group exhibited residual renal tubular structures, suggesting that the above fibrotic process was attenuated (zoom-in region). Under high-power magnification, evident epithelioid tubular-like cells were also visible in corresponding fibrotic regions in the HL/NS-398 group (zoom-in region), indicating tissue repair after inflammation. In contrast with the profound tubularinterstitial fibrosis in HL group, inhibitor-treated groups of NAC, AACOCF3 (AAC) and NS-398 attenuated such injury (yellow dotted line regions). The fibrosis area was marked by yellow dotted lines. Yellow arrows indicated the borderline of the fibrosis area. Scale bars are 2 mm, 1mm and 200 μm in the panels. (**B**) To compare the therapeutic effects of inhibitors, the HL/NAC and HL/NS-398 group presented the lowest degree of OKF after quantification analysis for the fibrotic area verses total tissue area (%). (**C**) After quantification analysis for the area of vacuoles, the HL/NAC group exerted the lowest degree of lipid vacuole deposition in the tubular epithelium. Quantification analysis was performed by image J. Data are expressed as mean ± SD (*n* = 8); * *p* < 0.05; and ** p < 0.01 to compare the differences between the two indicated groups. ns, not significant.

As we had already proven that HF and ME result in tubulointerstitial fibrosis, we aimed to investigate the underlying mechanism and inflammatory signaling pathways of OKF. Our previous research has contributed a mechanistic insight of uremic lung injury in a CKD mouse model, showing that ROS activates downstream PG pathways and recruits leukocytes to injured sites [25]. Furthermore, recent studies reported inhibitions of ROS and cPLA2/COX-2 ameliorates renal damages [10,11,12,13]. Nonetheless, therapeutic effects of the above inhibitors on OKF remain unclear. To investigate which factor mediated the progression of OKF, N-acetylcysteine (NAC; ROS scavenger), AACOCF3 (AAC; cPLA2 inhibitor), and NS-398 (COX-2 inhibitor) were used. Our results demonstrate significant fibrosis was found in juxtamedullary to medullar regions of HF and HL groups (Figure 3A). In corresponding fibrotic regions, the HL/NAC group exhibited residual renal tubular structures, suggesting that the above fibrotic process was attenuated (Figure 3A, zoom-in region). Under high-power magnification, evident epithelioid tubular-like cells were also seen in corresponding fibrotic regions in the HL/NS-398 group (Figure 3A, zoom-in region), indicating tissue repair after inflammation. In contrast with the severe tubularinterstitial fibrosis in the HL group, inhibitor-treated groups of NAC, AAC and NS-398 attenuated such injury (Figure 3A, yellow dotted line regions). To compare therapeutic effects of inhibitors, the HL/NAC and HL/NS-398 group presented the lowest degree of OKF after quantification analysis for fibrotic area verses total tissue area (%) (Figure 3B). Indeed, the results of Sirius Red stain confirmed that the HL groups contained the strongest red staining of fibrosis and treatment of NAC, AAC or NS398 alleviated the fibrotic staining (Supplementary Data 1). After quantification analysis to determine the area of vacuoles, the HL/NAC group exerted the lowest degree of LV deposition in the tubular epithelium (Figure 3C).

### 2.3. Scavengers of Non-Specific ROS Attenuate Not Only Oxidative Injury But Also Downstream Pathways of cPLA2 and COX-2 in Obese Kidney Fibrosis with Metabolic Endotoxemia

**Figure 4:**
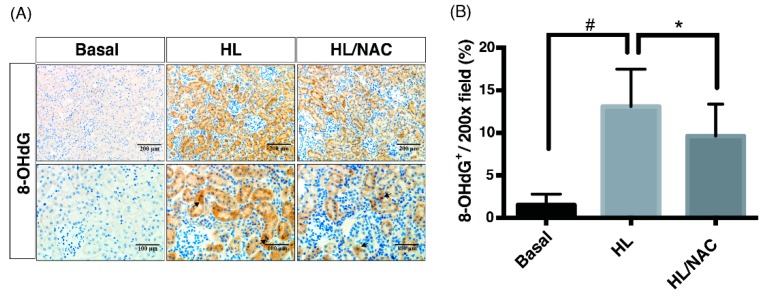
Scavengers of non-specific ROS attenuate oxidative injury in obese kidney fibrosis with metabolic endotoxemia. (**A**) Immunohistochemical staining methods were used to detect expressions of 8-OHdG. The HL group (HF mice with ME) exerts the highest expression of 8-OHdG, which was ameliorated by NAC. Scale bars are 200 μm or 100 μm in the panels. (**B**) Quantification analysis was performed by imageJ software. Data are expressed as mean ± SD (*n* = 8); * p < 0.05, *#* *p* < 0.01, to compare the differences between the two indicated groups.

**Figure 5:**
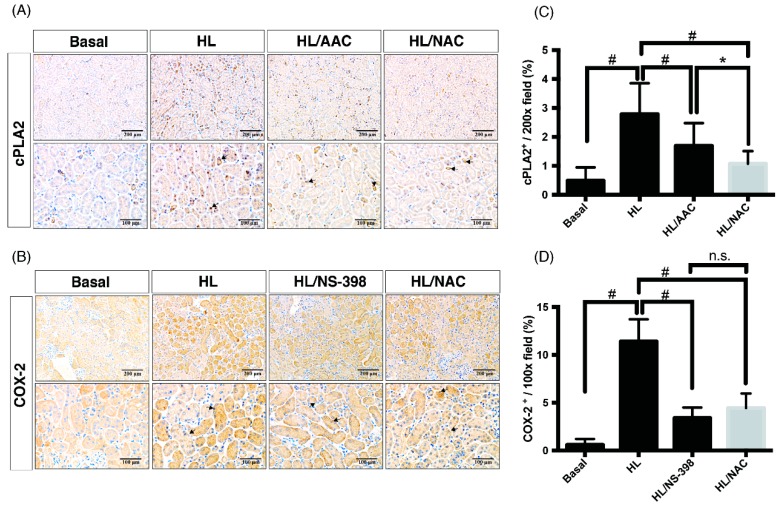
Scavengers of non-specific ROS reduced expression of cPLA2 and COX-2 in obese kidney fibrosis with metabolic endotoxemia. Immunohistochemical staining methods were used to detect expressions of (**A**) cPLA2, and (**B**) COX-2. The HL group (HF mice with ME) exerts the highest expression of cPLA2 and COX-2, which were ameliorated by NAC, AACCOCFS (AAC), and NS-398, respectively. Scale bars in the panels are 200 μm or 100 μm. (**C**,**D**) Quantification analysis indicated that ROS acts as the upstream key signal in the inflammatory cascades of OKF. Quantification analysis as performed by ImageJ software. Data are expressed as mean ± SD (*n* = 8); * p < 0.05, *#* *p* < 0.01, to compare the differences between the two indicated groups. n.s., not significant.

As we have proven, ROS, cPLA2 and COX-2 were involved in OKF with ME, the key messenger of signal transduction pathways in the OKF mechanism remain unclear. Given that ROS could function as a short-lived intracellular second messenger in signaling transduction [25,26,27], we hypothesized that ROS could function as an upstream signal transducer in cPLA2 and COX-2-mediated inflammatory pathways. To investigate it further, expressions of 8-hydroxy-2’-deoxyguanosine (8-OHdG), a derivative of oxidized deoxyguanosine, was evaluated as an indicator of oxidative damages. Results of IHC assay showed that there is an increased expression of 8-OHdG in the HL group (HF mice with ME) and treatment of NAC (scavenger of ROS) reduced the stain density of 8-OHdG in the HL/NAC group (Figure 4A,B). On the aspects of cPLA2 and COX-2 expression, as expected, the HL group (HF mice with ME) exerted the highest expression of cPLA2 and COX-2, which were ameliorated by AACOCF3 (AAC, inhibitor of cPLA2) and NS-398 (inhibitor of COX-2), respectively (Figure 5). Notably, the NAC-treated group ameliorated not only oxidative injury but also expressions of cPLA2 and COX-2 after the quantification analysis (Figure 5C,D), indicating that ROS acts as the upstream signal in the inflammatory cascades of OKF. Western blot was further used to confirm the results of IHC. As shown in the Supplementary Data 2, HL-induced expression of cPLA2 and COX-2 were attenuated by treatment of AAC, NAC or NS-398, separately. Collectively, ROS serves as a key pro-inflammatory signal to activate PG pathways, and ultimately, leads to OKF.

## 3. Discussion

In the present study, an OKF-ME mouse model was developed to demonstrate how a high-fat diet and LPS impair kidneys, providing a mechanical insight into OKF. Through testing the effects of ROS and cPLA2/COX-2 inhibitors, major breakthroughs were achieved, and the new findings markedly advance our understanding of OKF process.

### 3.1. The Fat–Intestine–Kidney Axis

The fat–intestine–kidney axis is the foundation stone of OKF, and underlying mechanisms are increasingly recognized through both human and animal models [4]. The role of an HF diet in promoting an obesogenic gut microbiota is undergoing confirmation, and gut dysbiosis can direct host storage of lipids in adipose tissue [28]. It has become more evident that the gut microbiota is altered in obesity, leading to activation of the LPS-toll-like receptor 4 (TLR4) axis and modulation of the intestinal barrier integrity [29]. Thus, intestinal dysbiosis in obesity has recently been recognized as a key environmental factor driving metabolic diseases, and ME via the increased paracellular transport of LPS is believed to contribute to chronic high-grade inflammation [30,31]. Studies in mice demonstrated that a HF diet or LPS infusion induced a two- to threefold increase in circulating LPS levels, contributing to the development of obesity and increased insulin resistance [32,33], and vice versa, LPS and TLR4 initiate a well-characterized signaling cascade that elicits intricate pro-oxidant and pro-inflammatory pathways in obesity [34]. A high LPS concentration is found in patients with type 2 diabetes [35], which is termed ME and reduced by the administration of hypoglycemic agents with potential anti-inflammatory effects [36]. Continuous administration of LPS resulted in recruiting inflammatory cells, activating mTOR signaling, tubular injury and collagen deposition in mice kidneys [37]. By contrast, the administration of antioxidants protects renal blood flow in LPS-treated animal models [38]. Recently, adipose-specific PLA2 have received attention for potential anti-obesity and anti-diabetic roles. Both genetic and pharmacological inhibition of particular PLA2s has resulted in obesity-resistant mouse models, suggesting a potential to develop new drugs [39]. In addition, COX-2 has long been believed to play a role in the inflammatory process as a result of obesity with an HF diet [9,40]. Given the role of PG pathways and oxidative stress in the etiology of obesity-induced renal injuries, the fat–intestine–kidney axis may intricately interact with ROS, cPLA2 and COX-2 in OKF progression. Current data suggest a healthy lifestyle—including antioxidant foods and the avoidance of excessive dietary fat intake—may ensure a friendly gut microbiota and positively affect prevention and treatment of various metabolic disorders.

### 3.2. Therapeutic Targets of ROS, cPLA2 and COX-2 in Kidney Diseases

Renal tubules are the main structure of the kidney and can be subjected to a variety of damage, including hypoxia, proteinuria, toxins, metabolic disorders, inflammation and oxidative stress [41]. Thus, tubular epithelial damage plays a pivotal role in renal fibrinogenesis [42,43], and PG pathways are crucial homeostatic modulators of kidney function [3]. Through the effects of cPLA2 in the initial stage, membrane phospholipids release arachidonic acid. In the following stepwise conversion of arachidonic acid by COX enzymes and PGE synthase, the major product of PGE2 is elevated and responsible for not only cardiovascular risks but also renal diseases [8]. Recent studies have shown that cPLA2 enhances proliferation and de-differentiation in human renal tubular epithelial cells [44], and silencing cPLA2 expression in knock-out (KO) mice is able to ameliorate pro-inflammatory eicosanoids production, inflammatory cell recruitment, and severity of fibrosis [16]. In another mouse model of high-carbohydrate high-fat-diet-induced obesity, the cPLA2 inhibitor treatment attenuated visceral adiposity and improved most features of metabolic syndrome, including insulin sensitivity, glucose intolerance, and cardiovascular abnormalities [10]. A considerable amount of literature reported that cPLA2 is a useful therapeutic target for diverse diseases. There are a number of concerns in using cPLA2 as a therapeutic target, especially because most of the studies are based on disease models comparing cPLA2 wild type (WT) and KO mice, which may not accurately reflect processes contributing to human diseases. This is apparent from the differences between mice and humans as a consequence of cPLA2 deficiency, e.g., cPLA2 is the first regulatory enzyme in the pathway for the production of numerous lipid mediators. Although, targeting cPLA2 may be beneficial in some diseases where COX metabolites contribute to diseases, such as asthma and arthritis [45]. However, PGs regulate labor and birth in humans, and an important source of PGs is amnion fibroblasts in fetal membranes [46,47]. Considering the essential roles of cPLA2 in human health, particularly for maintenance of the small intestine and female reproduction, is also a concern for targeting cPLA2 in OKF. Moreover, selective COX-2 inhibitors used in the rat model of an HF diet improved insulin sensitivity and TNFα mRNA expression [40]. Nonetheless, the nephrotoxic effect of COX-2 inhibitor for CKD patients is another concern in clinical practice.

Our research indicates ROS serve as a predisposition factor of OKF and thus a therapeutic target, activating downstream PG pathways and tubulointerstitial fibrosis. In contrast with PG inhibitors, NAC can easily be applied to a clinical therapeutic strategy, because a large oral dose of antioxidant is well tolerated without systemic side effects. Notably, the origin, the kinetics, and the localization of ROS generation all influence responses of T lymphocytes and inflammatory cells [48]. Shen Y et al. reported that the administration of NAC significantly mitigated oxidative and fibrotic responses resulting from angiotensin-II upregulation in the obstructed kidneys of mice, including expressions of fibronectin, collagen I, α-SMA and TGF-β [13].

We recognize several limitations of our study. In the first place, endogenous plasma creatinine levels and creatinine clearance as a tool to evaluate renal function were not evaluated in our mouse model. Difficulties have included the lack of an accurate, reproducible method to estimate renal function in conscious mice, problems obtaining sufficient blood volume and precisely timed urine collections repeatedly. Next, further parameters of tubulointerstitial injury in our OKF mouse models were not provided, e.g., neutrophil gelatinase-associated lipocalin, transforming growth factor beta, and alpha-smooth muscle actin. From the perspectives of nephrologists and pathologists, whereas the single method of Masson’s trichrome staining can easily and convincingly diagnose tubulointerstitial fibrosis in routine clinical practice of human renal biopsies.

**Figure 6:**
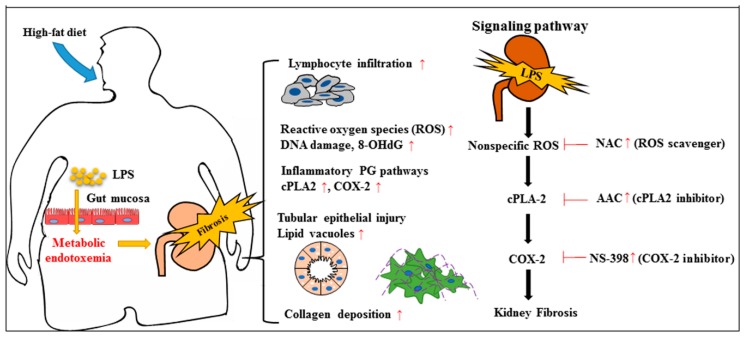
Potential mechanisms of obese kidney fibrosis induced by the fat–intestine–kidney axis. The schematic diagram has contributed a mechanistic insight into obese kidney fibrosis (OKF) with metabolic endotoxemia (ME). A high-fat diet leads to intestinal dysbiosis and hyperpermeability, favoring translocation of microbiome-derived lipopolysaccharide (LPS) to the bloodstream. A high-fat diet and ME impair kidneys through lipid deposition in the tubular epithelium, recruiting lymphocytes, triggering ROS to activate downstream prostaglandin pathways, and ultimately tubulointerstitial fibrosis. ROS may serve as predisposition factors and thus therapeutic targets in the prevention of OKF-ME. Different levels of the inhibitors’ activity are indicated as red arrows.

In conclusion, our research has contributed a mechanistic insight into OKF-ME, showing that a high-fat diet and ME impair kidneys through lipid deposition in the tubular epithelium, recruiting lymphocytes, triggering ROS to activate downstream PG pathways, and ultimately, tubulointerstitial fibrosis (Figure 6). ROS may serve as a predisposition factor of PG inflammatory pathways and OKF. We also elucidate that non-specific antioxidant NAC attenuates a high-fat diet and ME-induced renal inflammation and fibrosis. The protective effects are superior to cPLA2 and COX-2 inhibitors. This potential therapeutic target can easily be applied to clinical practice, because a large oral dose of NAC is well tolerated without systemic side effects. In light of the growing prevalence of obesity worldwide with an increasing trend in total medicare expenditures, the organ-protective effects of NAC should be tested in OKF patients who are in urgent need of new therapeutics. Several important issues in this research merit further discussion.

## 4. Materials and Methods

### 4.1. Materials

Most materials and methods were previously described [25]. Other materials utilized in this study can be purchased from Santa Cruz Biotechnology (Santa Cruz, CA, USA) or Sigma (St. Louis, MO, USA), including the inhibitor of cPLA2 (AACOCF3 (AAC)/Arachidonyltrifluoromethane), COX-2 (NS-398/N-[2-(cyclohexyloxy)-4-nitrophenyl] methanesulfonamide), and nonspecific ROS (NAC/N-Acetyl L-Cysteine).

### 4.2. Creating Animal Models to Mimic Obese Kidney Fibrosis (OKF) in Humans

A WT C57BL/6NCrlBltw mouse was provided by BioLASCO Taiwan Co., Ltd. (Taipei, Taiwan). The study was approved by the Animal Care and Use Committee of the Fu Jen Laboratory Animal Center (IACUC number: A10603; 1, March, 2017). All animals were handled according to the guidelines of the Animal Care Committee of Fu Jen Catholic University and NIH Guides for the Care and Use of Laboratory Animals. Animals were maintained in a temperature-controlled room (22 °C) under 12 h light–dark cycles. One week after arrival, four-week-old mice were divided into different groups. The control group received continuous feeding of a normal chow (Lactamin, Stockholm, Sweden). The HF group was first fed with a mix of a high-fat diet (#D12492, Research Diets, New Brunswick, NJ) and normal chow (*w*:*w* = 1:1) for diet adaptation for one week, then a complete high-fat diet course was given for the next 10 weeks. To create the animal model of OKF with ME, six-week-old mice that were fed with a high-fat diet were injected with LPS (10 μg/kg/week, intraperitoneal (i.p.) injection) as the HL group. If inhibitors were used, various inhibitors (2 mg/kg/week, i.p.) were injected one hour before LPS injection as inhibitor-treated groups. Mice were then sacrificed at the age of fifteen weeks, and kidneys were quickly removed and preserved in formalin for paraffin embedment for further analyses.

### 4.3. Tissue Preparation for Histopathological Evaluation of H&E Stain

Mice were anaesthetized via the inhalation of isoflurane and euthanized by cervical dislocation. Kidneys were removed and fixed in 10% formalin. Specimens were embedded in paraffin and sliced into 2–3 μm in thickness. Subsequently, the kidney tissues were stained with Hematoxylin-eosin (H&E stain). The images were captured using a Nikon Digital Camera Microscope (Nikon, Tokyo, Japan).

### 4.4. Masson’s Trichrome Staining Method

Masson’s trichrome staining method was used to determine the extent of collagen deposition and fibrosis in mouse kidney tissues. In the corresponding area, H&E staining of the adjacent paraffin section was performed for comparisons of tissue morphology. The experiments were conducted as follows: sections were first deparaffinized and rehydrated in ethanol/water solutions then post-fixed with Bouin’s solution for 1 h at room temperature. The fixation buffer was removed, and slides were stained with iron hematoxylin, Biebrich scarlet-acid fuchsin, and phosphomolybdic-phosphotungstic acid sequentially for 10 min per stain. Slides were then stained with Aniline blue. Finally, slides were washed in 10% acetate solution for 3–5 min and mounted in the mounting medium for observations to be made.

### 4.5. Immunohistochemistry Staining Method

Immunohistochemistry (IHC) was performed manually or automatically with an autostainer (BenchMark XT, Ventana Medical Systems Inc., Tucson, AZ, USA). For the manual protocol, paraffin sections were first deparaffinized and rehydrated in ethanol/water solutions. Epitopes on tissue were then retrieved with Heat-Induced Epitope Retrieval (HIER) in citrate buffer (0.01 M, pH 6.0). For blocking endogenous peroxidase activity, sections were treated with 3% hydrogen peroxide for 30 min in the dark. To reduce non-specific primary antibody binding, Blocking Buffer (DAKO) was used for one hour at room temperature. Sections were then incubated with primary antibodies at 4 °C overnight. Rabbit anti-mouse COX-2, anti-human cPLA2, and anti-8-OHdG polyclonal antibodies were purchased from Santa Cruz Biotechnology (SC-1747-R, SC-7891, and SC-139586, Santa Cruz, CA, USA). Afterward, staining was detected with a DAKO polymer system. For image acquisitions, three random high-power magnification fields were obtained in each sample by a Nikon Digital Camera Microscope (Nikon, Tokyo, Japan). All slides were reviewed by a blinded pathologist (Dr. Shih-Hao Liu), and the area percentage of staining in a 200× power magnification field were analyzed by NIH ImageJ software (Version 1.47, Bethesda, MD, USA).

### 4.6. Statistical Analysis of Data

All data are expressed as the mean ± SD using the GraphPad Prism Program (GraphPad, San Diego, CA, USA). Quantitative data were analyzed with a non-paired Student’s t-test. The significance threshold was set at 5% (*p* < 0.05). All of the experiments were performed at least three times.

## Supplementary Materials

The following are available online at https://www.mdpi.com/1422-0067/20/18/4393/s1.

## Abbreviations

CKD	chronic kidney disease
COX-2	cyclooxygenase-2
cPLA2	cytosolic phospholipases A2
HF group	high-fat diet-fed group injected with lipopolysaccharide
IHC	immunohistochemistry
LPS	lipopolysaccharide
ME	metabolic endotoxemia
OKF	obese kidney fibrosis
PG	prostaglandin
ROS	reactive oxygen species
