# Supplementary material for: Targeting ROS and cPLA2/COX2 Expressions Ameliorated Renal Damage in Obese Mice with Endotoxemia
Source: Int J Mol Sci. 2019 Sep 6;20(18):4393. doi: 10.3390/ijms20184393 (PMC6769974; doi:10.3390/ijms20184393)
Supplement: Supplementary file 1 [file ijms-20-04393-s001.pdf]

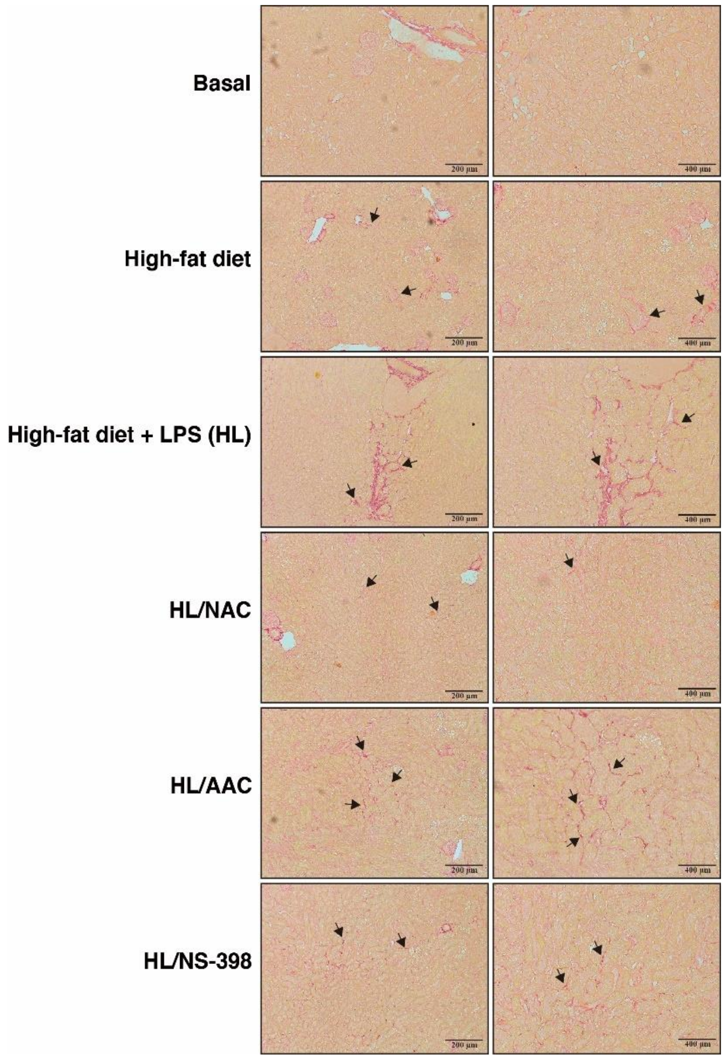

Supplemental data 1. Inhibitors of ROS, cPLA2, and COX-2 attenuated the fibrosis in HF mice with ME. (A) At the end of treatment, mice were scarified and kidney tissues were extracted for Sirius Red Stain. Compared with the normal chow-fed control group (basal group) and high-fat diet-fed group (HF group) mice, high-fat diet-fed group with lipopolysaccharide (LPS) treatment (HL group) exhibited the most prominent renal fibrosis. Pretreatment of NAC (ROS scavenger), AACOCF3 (AAC; cPLA2 inhibitor) and NS-398 (COX-2 inhibitor) reduced the staining of fibrotic region. Black arrows indicate the positive stain of Sirius Red.

(A)

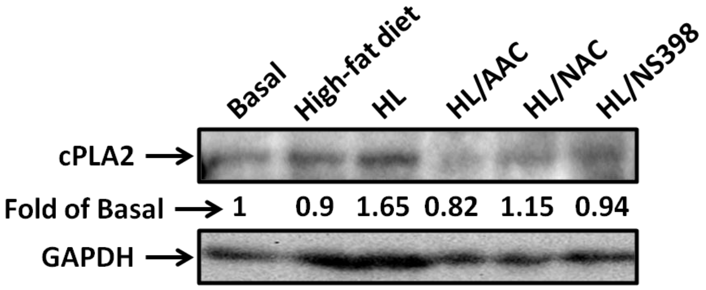

(B)

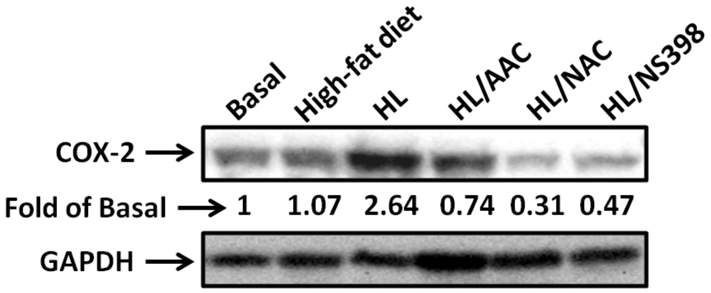

Supplemental data 2. Inhibition effects of AAC, NAC and NS-398 on HL-related cPLA2 and COX-2 protein expression. Proteins were extracted from the residual kidney tissues of various groups. After quantification and normalization, protein samples were subjected into SDS-PAGE with 10 % running gel. Western blot was performed with (A) anti-cPLA2, or (B) anti-COX-2 antibodies. Membranes were stripped and re-probed with anti-GAPDH antibody as internal control.

Supplemental Methods

Supplemental Methods 1. Sirius Red staining method

Sirius Red staining method was used to determine the extent of collagen deposition in mouse kidney tissues. Briefly, the tissue sections were first deparaffinized and rehydrated in ethanol/water solutions. And then adequate Picro-Sirius Red solution was applied to the tissue section for 60 minutes. After acetic acid and absolute alcohol rinsed, slides were cleared and mounted for observations. Collagen components were in red-stained and non-collagen components were in orange-stained, respectively.

Supplemental Methods 2. Western blot analysis of cPLA2 and COX-2 expression

Protein samples were extracted from the residue renal tissues of various groups. Proteins were quantified and subjected to 10% SDS-PAGE. Proteins were transferred to nitrocellulose membrane, and the membrane was incubated successively at room temperature with 5% BSA in Tris-buffered saline with 0.1% Tween 20 (TTBS) for 1 h. Membranes were incubated overnight at 4°C with an anti-cPLA2, anti-COX-2, or anti-GAPDH according to the recommendation of the manufacturer. Anti-COX-2 (SC-1745) and anti-cPLA2 (sc-454) antibodies were purchased from Santa Cruz Biotechnology (CA, USA). Anti-GAPDH antibody (#2118) was purchased from Cell Signaling Technology (MA, USA). Membranes were incubated with a 1:2,000 dilution of anti-goat, anti-mouse or anti-rabbit horseradish peroxidase antibody for 1 h. The immunoreactive bands detected by ECL reagents were developed by Hyperfilm-ECL.
